# Appraising Drugs Based on Cost-effectiveness and Severity of Disease in Norwegian Drug Coverage Decisions

**DOI:** 10.1001/jamanetworkopen.2022.19503

**Published:** 2022-06-29

**Authors:** Eirik Joakim Tranvåg, Øystein Ariansen Haaland, Bjarne Robberstad, Ole Frithjof Norheim

**Affiliations:** 1 Bergen Centre for Ethics and Priority Setting, Department of Global Public Health and Primary Care, University of Bergen, Bergen, Norway; 2 Centre for Cancer Biomarkers, Department of Global Public Health and Primary Care, University of Bergen, Bergen, Norway; 3 Research Group in Health Economics, Leadership, and Translational Ethics Research, Department of Global Public Health and Primary Care, University of Bergen, Bergen, Norway

## Abstract

**Importance:**

Rising health care costs are a major health policy challenge globally. Norway has implemented a priority-setting system intended to balance cost-effectiveness and concerns for fair distribution, but little is known about this strategy and whether it works in practice.

**Objective:**

To present and evaluate a systematic drug appraisal method that uses the severity of disease to account for a fair distribution of health in cost-effectiveness analysis, forming the basis for price negotiations and coverage decisions.

**Design, Setting, and Participants:**

This cross-sectional study uses confidential drug price information and publicly available data from health technology assessments and logistic and linear regression analyses to evaluate drug coverage decisions for the Norwegian specialized health care sector from 2014 to 2019.

**Main Outcomes and Measures:**

Drug coverage decisions by Norwegian authorities and incremental cost-effectiveness and severity of disease measured as absolute shortfall of quality adjusted life years.

**Results:**

Between 2014 and 2019, a total of 188 drugs were appraised, of which 113 were cancer drugs. The overall coverage rate was 73% (138 of 188). The number of annual appraisals increased during the observation period. Based on 83 chosen decisions, regression analysis showed that incremental cost-effectiveness ratios (ICER) based on negotiated drug prices, adjusted for severity-differentiated cost-effectiveness thresholds, was the variable that best projected drug approvals (OR, 0.60; 95% CI, 0.42-0.86). An increase in the ICER by $10 000 was associated with a reduction in the odds for approval of 40% for drugs assessed from 2018 to 2019.

**Conclusions and Relevance:**

This cross-sectional study demonstrated how concerns for efficiency and fair distribution of health can be implemented systematically into drug appraisals and reimbursement decisions. New, expensive drugs are expected to escalate health care costs in the years to come, and it may be feasible to control costs by negotiating the prices of new drugs while appraising both their cost-effectiveness and how their health benefits are distributed.

## Key Points

**Question:**

How are drug coverage decisions within the Norwegian specialist health sector made, and are they in accordance with the decided principles of cost-effectiveness and fair distribution of health outcomes?

**Findings:**

In this cross-sectional study of 83 decisions made in Norway between 2014 and 2019, the incremental cost-effectiveness ratio (ICER) based on negotiated drug prices, adjusted for severity-differentiated cost-effectiveness thresholds, best projected drug approval.

**Meaning:**

These findings suggest that cost-effectiveness and fair distribution of health outcomes are systematically implemented in the Norwegian drug approval system, and including these principles may be a feasible strategy for controlling increasing drug costs.

## Teaser

This cross-sectional study evaluates a systematic drug appraisal method that uses severity of disease to account for a fair distribution of health in cost-effectiveness analysis.

## Introduction

The rising cost of health care is a major challenge for health policy, and there is an ongoing debate about how these costs can be contained fairly.^1,2,3^ Cancer drugs are seen as a key driver of increasing drug costs.^4,5,6,7^ One favored strategy to control drug prices is through price negotiations and appraisals of their cost-effectiveness.^8,9^ Such concerns for efficacy is motivated by ethical arguments.^10,11^ A legitimate critique of this health maximizing approach is that the distribution of gains and losses for the worse off in health is ignored.^12,13^ A concern for fair distribution of health is important because priority setting decisions motivated solely by health maximization can increase health inequalities.

In Norway, this concern for fair distribution is addressed by integrating a systematic assessment of disease severity into coverage decisions for new drugs.^14,15^ The severity criterion gives extra priority to patients for whom health care benefits matter more (eg, patients with severe chronic conditions or patients living with disabilities).^16^ Appraisals are guided by 3 priority setting criteria: health benefit, resource use, and disease severity.^17^ Patients with more severe disease have a stronger claim to treatment and, therefore, everything else being equal, are given higher priority.

This analysis aims to present and evaluate a drug appraisal system that balances concerns for health maximization and fair distribution of health outcomes in an explicit and systematic manner. Using unique cost-effectiveness data on confidentially negotiated drug prices, we investigated whether drug coverage decisions for publicly funded Norwegian hospitals are made in accordance with Norway’s priority-setting criteria. These confidential prices provide a rare insight into the decision basis for drug coverage decisions. Special consideration is given to cancer drugs to inform the debate about how health systems can evaluate and cover costly new cancer drugs.

## Methods

Data used in this cross-sectional study is impersonal and not linked to any patients. Therefore, neither ethical review nor informed consent was necessary. This study followed the Strengthening the Reporting of Observational Studies in Epidemiology (STROBE) reporting guideline for cross-sectional studies.

### A System for Priority Setting and Drug Coverage Decisions

In Norway, coverage decisions for new drugs are made by the National System for Managed Introduction of New Health Technologies within the Specialist Health Service (New Methods System) based on 3 criteria for priority setting^18^: health benefit, resource use, and severity of disease (eTable 1 in the Supplement). To make decisions, the criteria are balanced and weighed against each other. For example, if a condition is considered to be very severe, or if a treatment provides large health benefits compared with the current standard of care, greater resource use can be acceptable. In practice, and with special relevance for the drug approval system, the health benefit and resource criteria are assessed together as a cost-effectiveness criterion. The incremental cost-effectiveness ratio (ICER) is the difference in costs between the new treatment and the current standard of care divided by the difference in effect between them.

Fair distribution of health gains is implemented by giving extra priority to patient groups with larger loss of prospective health (ie, low health-adjusted life expectancy), emphasizing a key dimension of the severity of a disease. In Norway, disease severity is defined as the mean absolute shortfall of quality-adjusted life-years (QALYs) in the patient group receiving standard care compared with a reference group of healthy persons of a similar age.^17,19,20^ Absolute shortfall was formally introduced into drug reimbursement decisions January 1, 2018. A higher threshold for willingness-to-pay per QALY by the government is accepted for interventions treating more severe disease groups. eTable 2 in the Supplement presents the 6 classes of severity suggested by a public commission with corresponding cost-effectiveness thresholds.^21^

After a drug has been granted a marketing license, manufacturers can apply for coverage. Coverage decisions for single drugs are usually based on Health Technology Assessments, where evidence on clinical benefit, cost-effectiveness, and budget impact submitted by drug manufacturers are complemented with input from independent clinical experts and revised by health economists at the Norwegian Medicines Agency. There are publicly available guidelines for these assessments that describe the economic evaluations, QALY estimations, sensitivity analysis, and other elements.^22^ These guidelines aim to ensure that all drugs are consistently appraised. If a drug is approved, the drug is covered for all eligible patients in the national health care system. If it is declined (which most often happens because of an unfavorable ICER), price negotiation with the manufacturer is mandated. This negotiation may result in a more favorable ICER and, depending on the size of the price discount, a subsequent approval. The negotiated prices and related information that may be used to elicit them, such as information about cost-effectiveness and budget impact, are subject to confidentiality.

### Data

We obtained lists of all coverage decisions made from 2014 to 2019, each of which was assessed and revised as illustrated in eFigure 1 in the Supplement. For each of the 188 decisions identified for further analysis, information about drug name, disease type, indication, ICER based on publicly-available list price, severity of disease, biomarker status, and decision outcome (approved or refused) were extracted from published technology assessments.^23^ Revised ICERs based on confidential price negotiations were obtained from the secretariat at the New Methods System after a formal application. Access to the data was granted based on §13d of the Public Administration Act, on the condition that the authors do not reveal (1) individual confidential drug prices or (2) the government’s cost-effectiveness thresholds.

### Statistical Analysis

All analyses were performed with R version 4.0.3 (R Project for Statistical Computing). Descriptive statistics on decisions per year and approval ratios were calculated based on all 188 decisions. Further analyses were performed on the 83 decisions with complete information about ICERs based on negotiated drug prices and severity of disease (eFigure 1 in the Supplement). Many drugs were excluded because they did not go through a full cost-effectiveness analysis, typically because simplified assessments, such as cost-minimization analysis or summaries of effect, safety, and costs, were considered adequate. Importantly, drugs with new indications or high expected price do typically go through full cost-effectiveness analysis.

Two sets of regression analyses were performed. In the first set of analyses, we used logistic regression with coverage decision as the outcome variable and cost-effectiveness as the explanatory variable. We used 3 measures of cost-effectiveness: (1) ICER based on publicly available drug prices (public ICER), (2) ICER based on negotiated drug prices (negotiated ICER), and (3) negotiated ICER adjusted for severity-differentiated cost-effectiveness thresholds. Analyses were performed separately for the period before (2014-2017, n = 40) and after (2018-2019, n = 43) the specification of severity in coverage decisions was implemented and pooled for both periods. In the second set of analyses, we used linear regression with negotiated ICERs as the outcome and severity of disease (absolute QALY shortfall) as the explanatory variable, performing analyses for 2014 to 2017 and 2018 to 2019 separately and stratifying by whether or not the drug was approved.

Based on weights as listed in eTable in the Supplement, we calculated severity-adjusted ICERs as follows. Let *x* denote the severity of a condition according to the 6 categories, *w(x)* the weight of the severity category, and ICER_1_ the ICER threshold in category 1. We now have that severity-adjusted ICER = ICER – (w[x]-1) × ICER_1_. Analysis was performed in April 2022, and data were collected from December 2020 to January 2021.

## Results

This study included 188 total coverage decisions for all drugs, 75 (40%) of which were for non–cancer drugs and 113 (60%) were for cancer drugs. The number of coverage decisions has increased over time from 8 in 2014 to 68 in 2019 (Table 1). For cancer drugs, the approvals varied from 12 of 12 decisions (100%) in 2015 to 4 of 9 decisions (44%) in 2016, with a total approval 82 of 113 (73%). Cancer drugs were the most frequently appraised drugs, representing 113 of 188 (60%) of decisions in the period. The overall approval ratio for cancer drugs was equal to the overall ratio for all drugs.

**Table 1.** Overview of All Drug Coverage Decisions, 2014-2019

Year	All drugs^a^		Non–cancer drugs		Cancer drugs^b^	
	Total decisions	Approvals, No. (%)	Total decisions	Approvals, No. (%)	Total decisions	Approvals, No. (%)
2014	8	5 (63)	1	1 (100)	7	4 (57)
2015	16	16 (100)	4	4 (100)	12	12 (100)
2016	15	8 (53)	6	4 (67)	9	4 (44)
2017	37	33 (89)	15	15 (100)	22	18 (82)
2018	44	23 (52)	17	7 (41)	27	16 (59)
2019	68	53 (78)	32	25 (78)	36	28 (78)
Total	188	138 (73)	75	56 (75)	113	82 (73)

^a^ All drugs include all drugs for all indications.

^b^ Cancer drugs include all drugs with a cancer-type indication.

The logistic regression analysis in Table 2 shows that the severity-adjusted ICER best projected a positive drug coverage decision. The lowest odds ratio was for decisions made in 2018 to 2019 when severity differentiation was formally introduced in the system (OR, 0.60; 95% CI, 0.42-0.86). We also found a strong effect size for negotiated ICER (OR, 0.71; 95% CI, 0.58-0.86). Projections from the public ICER were weak (OR, 0.92; 95% CI, 0.86-0.98).

**Table 2.** Regression Analysis of Coverage Decision^a^

ICER	All decisions			2014-2017			2018-2019		
	OR (95% CI)	*R* ^2^	AIC	OR (95% CI)	*R* ^2^	AIC	OR (95% CI)	*R* ^2^	AIC
Public^b^	0.92 (0.86 to 0.98)	0.06	104.6	0.92 (0.84 to 1)	0.07	50.0	0.92 (0.83 to 1)	0.04	58.0
Negotiated^c^	0.71 (0.58 to 0.86)	0.23	82.4	0.74 (0.58 to 0.94)	0.27	38.7	0.64 (0.45 to 0.9)	0.24	44.5
Severity-adjusted^d^	0.68 (0.54 to 0.85)	0.24	77.4	0.69 (0.51 to 0.93)	0.31	32.7	0.60 (0.42 to 0.86)	0.27	42.8

Abbreviations: AIC, Akaike information criterion; ICER, incremental cost-effectiveness ratio; OR, odds ratio; *R*^2^, McFadden *R*^2^.

^a^ The outcome variable is approved. ORs for ICERs are per $10 000/QALY. Each model only included 1 of the ICER-variables (public ICER, negotiated ICER, or severity-adjusted ICER). AIC values can only be compared within the same data set (ie, within each column). Low values indicate a better fit to the data. A difference of 2 between models is considered substantial. The relative difference between 2 AIC values is irrelevant.

^b^ Public ICERs are based on publicly available prices for drugs.

^c^ Negotiated ICERs are based on the negotiated drug prices.

^d^ Severity-adjusted ICERs are based on negotiated drug prices adjusted for severity-differentiated cost-effectiveness thresholds (see the equation in the Methods section).

**Figure 1.**
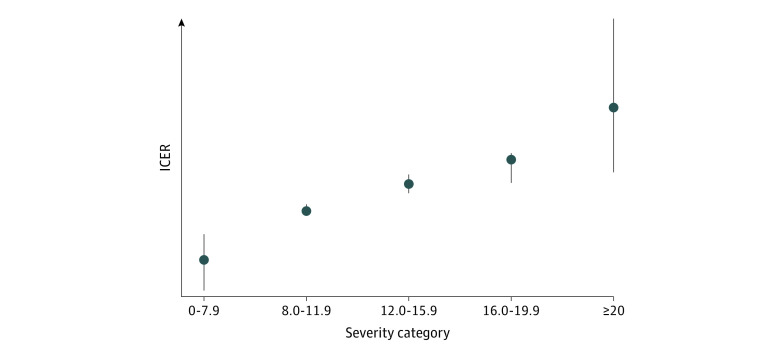
Incremental Cost-effectiveness Ratios (ICER) for Drugs Approved for Coverage in 2018 and 2019 Negotiated ICERs for approved drugs in 2018 and 2019 plotted from the 25th (bottom) to 75th (top) percentile values with medians and grouped by severity of disease as measured by absolute quality-adjusted life-year shortfall. The 2 lowest-severity categories (0-4 and 4-8) are pooled because of the low number of observations. To keep individual ICERs confidential, only the quartiles and median values are plotted. The distance between groups on the y-axis indicates the absolute difference in ICER between the groups, the dots indicate the median, and the error bars indicate the IQR.

We found a strong negative association between ICER and the probability of approval after price negotiations and severity of disease were taken into account. When the ICER increased by $10 000, the odds for approval dropped by 40% for coverage decisions made in 2018 to 2019. This trend is also demonstrated in eFigure 2 in the Supplement. We found a strong association between severity in terms of QALY loss and negotiated ICER for approved drugs between 2018 and 2019, implying that higher ICERs were indeed accepted for drugs treating more severe diseases. For the period of 2014 to 2017, this association was much weaker. Figure 1 shows the approved drugs in 2018 to 2019 grouped into severity classes, and illustrating that higher ICERs were more frequently accepted in the highest severity category.

**Figure 2.**
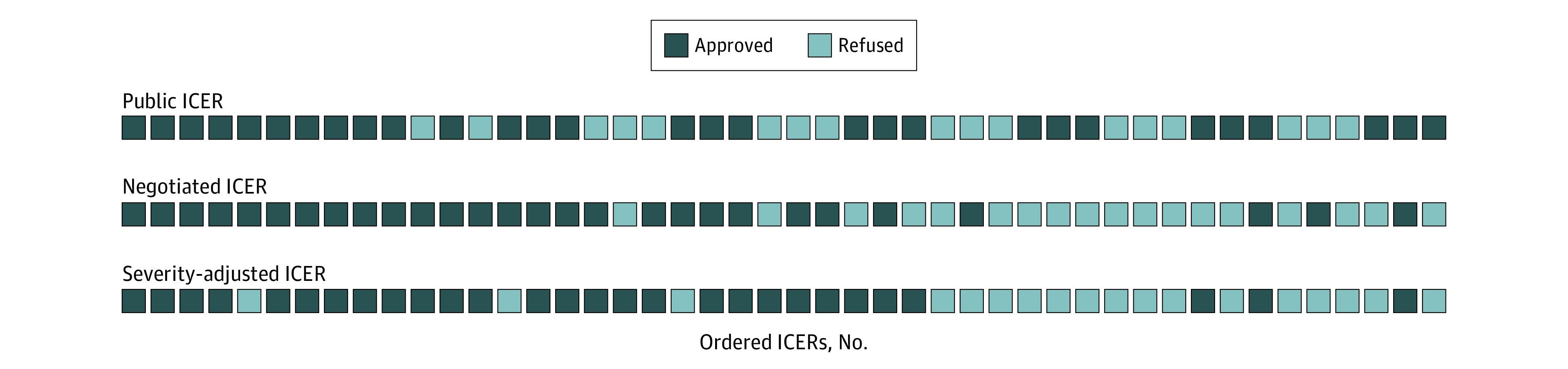
Drug Coverage Decisions in 2018 and 2019 Ordered by Increasing Incremental Cost-effectiveness Ratios (ICERs) The colored boxes represent the 45 drug-approval decisions made in 2018 and 2019 (28 drugs approved and 17 drugs were rejected) but with the ICER calculated based on different criteria. Public ICERs were based on publicly available list prices for drugs. Negotiated ICERs were based on negotiated drug prices. Severity-adjusted ICERs were based on negotiated drug prices, adjusted for severity-differentiated cost-effectiveness thresholds (see the equation in the Methods section).

When all drugs appraised in 2018 and 2019 were sorted by increasing ICER, patterns of approval and rejection changed according to how the ICERs were estimated. Figure 2 visualizes the regression analysis in Table 2 and demonstrates that the likelihood of approval was more strongly associated with severity-adjusted and negotiated price ICERs than with public ICERs. With negotiated ICERs and severity-adjusted ICERs, most drugs with low ICERs were approved while most drugs with high ICERs were refused. The 3 drugs approved with a high ICER were all given extra priority because they were treatments for rare diseases.

**Figure 3.**
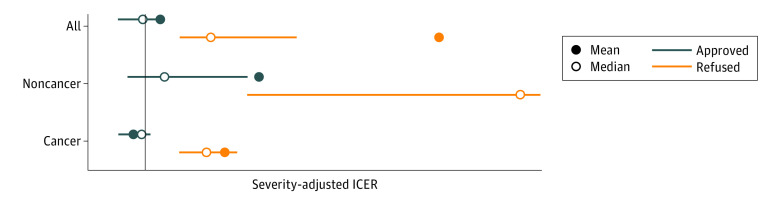
Severity-Adjusted Incremental Cost-effectiveness Ratios (ICER) for Approved and Rejected Drugs in 2018 and 2019 Severity-adjusted ICER for approved and refused drugs plotted as the 25th (left) to 75th (right) percentile values, with means and medians. Because not all values are visualized here, mean and median values may fall outside of the lines. The vertical line indicates the highest cost-effectiveness threshold for the lowest severity group and was used to adjust all ICERs. All indicates all drugs irrespective of disease; cancer, drugs treating cancer; noncancer, drugs treating diseases other than cancer.

Approved drugs had a much lower ICER than rejected drugs (Figure 3). Most approved cancer drugs had an ICER below the severity-adjusted cost-effectiveness threshold, while several approved non–cancer drugs had an ICER well above the threshold. Also, the high ICERs for rejected non–cancer drugs were striking.

## Discussion

The findings of this study suggest that cost-effectiveness estimates are systematically adjusted with concerns for fair distribution of health gains in appraisals for new drugs in Norway. By analyzing a unique data set based on confidentially negotiated drug prices, we found an association between price negotiations, cost-effectiveness estimates, and drug coverage decisions. We observed a strong negative association between ICERs and the probability of approval after price negotiations and fair distribution of health gains were taken into account, and a much weaker association of public ICERs based on list prices, which is the nonconfidential and uncensored information available to the public. These findings suggest that giving priority according to severity of disease is implemented in practice.

Severity of illness is 1 dimension of fairness, or health equity, that may be seen as relevant in priority setting decisions and that may be weighted against cost-effectiveness.^24^ Socioeconomic status, ethnicity, geography, and age are examples.^25^ In Figure 2, another dimension of fairness is seen, namely priority to rare diseases. In the line where 45 decisions are ordered by their severity-adjusted ICERs, 3 purple boxes illustrating 3 approved drugs are located far to the right, meaning that their incremental cost-effectiveness ratios were far higher than normally accepted. For very rare diseases with a high severity, Norway appears to accept a higher resource use and lower quality of evidence.^18^ These 3 decisions may also explain the high ICERs for approved non–cancer drugs in Figure 3.

Many countries apply cost-effectiveness analysis in technology appraisals to link therapeutic added value and incremental cost to drug coverage decisions,^26,27^ but few incorporate concerns for fair distribution. It has been shown in the UK,^28^ Canada,^29^ Sweden,^30^ and Australia^31^ that cost-effectiveness informs drug coverage decisions, but only Sweden, the Netherlands, and the UK systematically incorporate concerns for fair distribution of health.

Sweden uses severity of disease to differentiate among various cost-effectiveness thresholds, but the criterion is not clearly defined and lacks a standardized approach for assessment.^20,30^ In the Netherlands, severity of illness is formally and systematically part of decisions with 3 different ICER thresholds depending on proportional QALY shortfall.^32^ In the UK, severity of illness is part of The National Institute for Health and Care Excellence’s (NICE) social value judgements, but illness severity has not been used to weigh QALYs.^33^ NICE has been using an end-of-life criterion, which sets a higher cost-effectiveness threshold for treatments used toward the end of life, but this custom is not justified by a concern for fair distribution.^34^ However, a new methods manual for NICE’s health technology assessments will replace the end-of-life criterion with a severity modifier, meaning that higher priority should be given to treatments with larger absolute or proportional QALY shortfall.^35^ This new method is to give incremental QALY gains from new treatments higher weight if the disease severity is sufficiently large.^36^

With so many countries using cost-effectiveness in their decisions, it is not surprising that price negotiations are common.^37,38^ As our analyses suggest, price negotiations where the buyer directly negotiates with the drug manufacturer can be a powerful method for lowering drug prices, thus making ICERs more favorable, which consequentially increases the likelihood for approval.

Negotiated drug prices are typically confidential, which raises ethical concerns. First, when publicly available information about list prices for drugs do not reflect actual prices, the comparison of drug prices across countries becomes more difficult and less relevant.^39,40^ Another ethical concern is that confidential pricing reduces transparency and legitimacy in the health care system. This lack of procedural fairness makes it difficult for the public to assess whether the criteria are adhered to in practice and to what degree access to new treatments is equitable and fair. This problem is highlighted by our finding that public ICERs were not associated with priority decisions. Price confidentiality may reduce drug prices and result in more patients getting access to new drugs, but such claims lack empirical support in Norway. Another important questions is if the cost-effectiveness thresholds used in the appraisals have empirical support in Norway. In short, balancing procedural and consequentialist aspects of fairness is a challenge that requires further economical, ethical, and legal research.

### Limitations

This study had limitations. One limitation of our analysis is that 95 of the 188 decisions were appraised without updated estimates of ICER. Instead, simplified assessments were performed to make the appraisal process more effective. These 95 decisions should in principle also be made in accordance with the priority setting criteria, but it was not possible to include them in our analysis because no ICER was estimated. Also, information about budget impact and uncertainty of evidence were not systematically reported in a quantifiable manner and were, therefore, difficult to extract from the reports.

Lastly, the 2 restrictions on access given to the confidential data (based on §13 of the Public Administration Act^41^) prevented us from publishing more detailed analyses and figures. These restrictions, together with systematic redaction of published documentation from evaluations and meetings, made it impossible to provide full insight into decisions and rationales. The 3 coverage decisions in Figure 2 that were refused despite a relatively low severity-adjusted ICER are examples of this. We were unable to extract any clear reason for their refusal from the publicly available information. This is a matter of fair process. It is widely acknowledged that transparency in public rationing decisions is crucial for a legitimate process and trust in the system.^42^ Even given these limitations, we believe that our analyses bring valuable insight and knowledge about an important topic in health policy, namely priority setting, for new drugs.

## Conclusions

In this study, higher ICERs were associated with lower odds of approval after price negotiations and severity of disease were taken into account. This suggests how cost-effectiveness, price negotiations, and concerns for a fair distribution of health gains are implemented in the Norwegian drug appraisal system, and appraising drugs based on these principles of fairness is a feasible strategy for controlling increasing drug costs.

## Notes

Supplement. **eTable 1.** Norway and Its Health Care System **eTable 2.** Differentiated Cost-Effectiveness Thresholds According to Severity Category **eFigure 1.** Flow Chart Illustrating Inclusion and Exclusion of Drug Reimbursement Decisions for Our Analysis **eFigure 2.** Plot of Drug Coverage Decisions for 2014 to 2017 and 2018 to 2019
eReferences.
