# Supplementary material for: Appraising Drugs Based on Cost-effectiveness and Severity of Disease in Norwegian Drug Coverage Decisions
Source: JAMA Netw Open. 2022 Jun 29;5(6):e2219503. doi: 10.1001/jamanetworkopen.2022.19503 (PMC9244608; doi:10.1001/jamanetworkopen.2022.19503)
Supplement: Supplement. — eTable 1. Norway and Its Health Care System eTable 2. Differentiated Cost-Effectiveness Thresholds According to Severity Category eFigure 1. Flow Chart Illustrating Inclusion and Exclusion of Drug Reimbursement Decisions for Our Analysis eFigure 2. Plot of Drug Coverage Decisions for 2014 to 2017 and 2018 to 2019 eReferences. [file jamanetwopen-e2219503-s001.pdf]

Supplemental Online Content

Tranvåg EJ, Haaland ØA, Robberstad B, Norheim OF. Appraising drugs based on cost-effectiveness and severity of disease in Norwegian drug coverage decisions. *JAMA Netw Open*. 2022;5(6):e2219503. doi:10.1001/jamanetworkopen.2022.19503

eTable 1. Norway and Its Health Care System
eTable 2. Differentiated Cost-Effectiveness Thresholds According to Severity Category
eFigure 1. Flow Chart Illustrating Inclusion and Exclusion of Drug Reimbursement Decisions for Our Analysis
eFigure 2. Plot of Drug Coverage Decisions for 2014 to 2017 and 2018 to 2019
eReferences

This supplemental material has been provided by the authors to give readers additional information about their work.

© 2022 Tranvåg EJ et al. *JAMA Network Open.*

eTable 1. Norway and Its Health Care System[1,2]

- Population of 5.3 million
- Single-payer tax-based health care system
- Public health care spending is 85% of total health care spending
- Per-capita health care expenditure of $6187
- Three priority-setting criteria are applied in the health care system:
    - The health-benefit criterion: the priority of an intervention increases with the expected health benefit from the intervention
    - The resource criterion: the priority of an intervention increases the less resources it requires
    - The severity criterion: the priority of an intervention increases with increasing severity of the condition the intervention is targeting

References

1. Ord T. The moral imperative toward cost-effectiveness in global health. In: Effective Altruism. *Oxford University Press*; 2013.
2. Neumann PJ, Cohen JT, Ollendorf DA. Drug-pricing debate redux—should cost-effectiveness analysis be used now to price pharmaceuticals? *N Engl J Med*. 2021;385(21):1923-1924. Medline:34767318 doi:10.1056/NEJMp2113323

© 2022 Tranvåg EJ et al. *JAMA Network Open.*

eTable 2. Differentiated Cost-Effectiveness Thresholds According to Severity Category

**Severity category (x)**	**1**	**2**	**3**	**4**	**5**	**6**
Absolute QALY shortfall	0–3.9	4–7.9	8–11.9	12–15.9	16–20	> 20
ICER Threshold ($/QALY)	32 000	45 000	58 000	71 000	84 000	97 000
Weight (w)	1.0	1.4	1.8	2.2	2.6	3.0

Differentiated cost-effectiveness thresholds according to severity category, as proposed by Magnussen et al.[1] The lower category 1 threshold is an approximation from Klaxton et al.[2] The other thresholds were suggested, after a review of surveys of inequality aversion, by the committees preparing the white paper. Drugs are classified into severity categories based on the estimated severity of the disease they target, measured as absolute QALY shortfall. For each category an upper ICER threshold is suggested. The weight is the relationship between the thresholds.

© 2022 Tranvåg EJ et al. *JAMA Network Open.*

eFigure 1. Flow Chart Illustrating Inclusion and Exclusion of Drug Reimbursement Decisions for Our Analysis

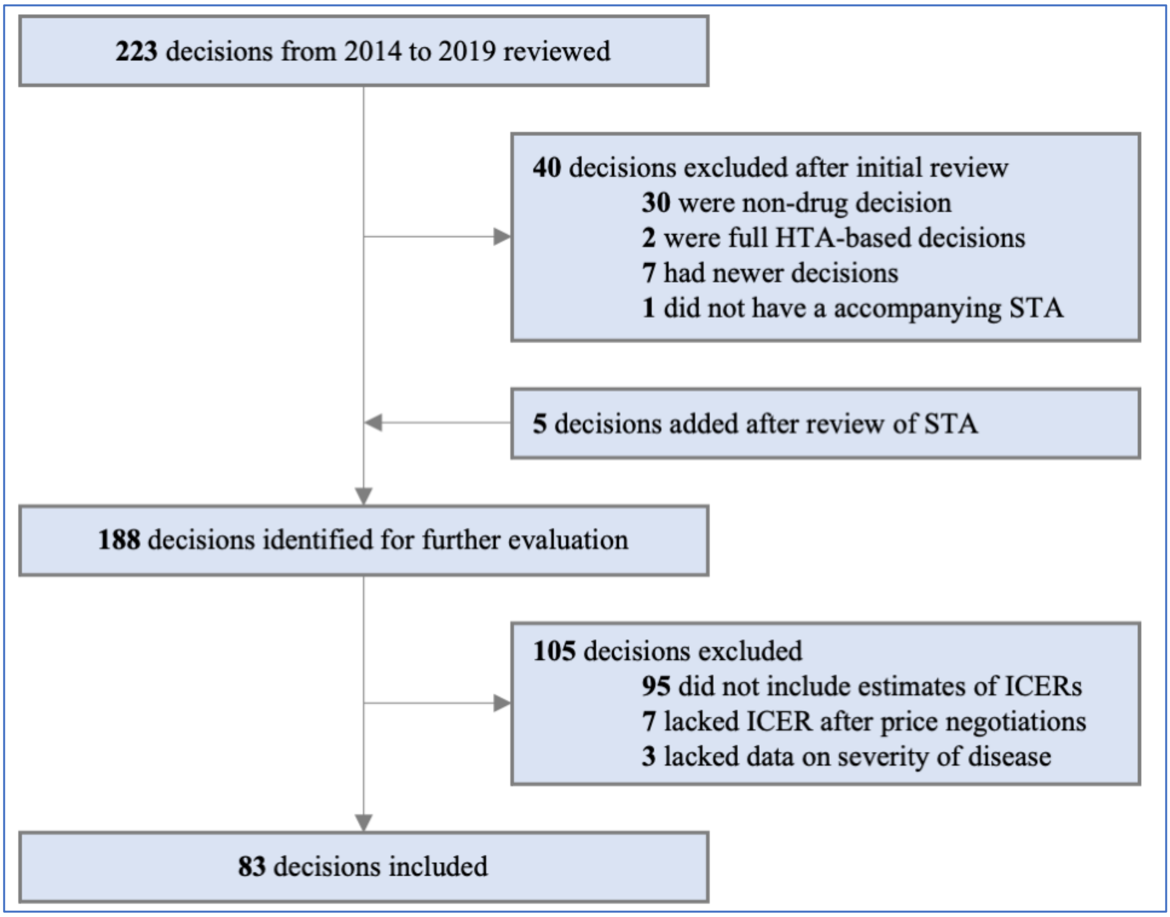

Figure legend: STA = Single Health Technology Assessment, HTA = Health Technology Assessment, ICER= Incremental Cost-Effectiveness Ratio

© 2022 Tranvåg EJ et al. *JAMA Network Open.*

eFigure 2: Plot of Drug Coverage Decisions for 2014 to 2017 and 2018 to 2019

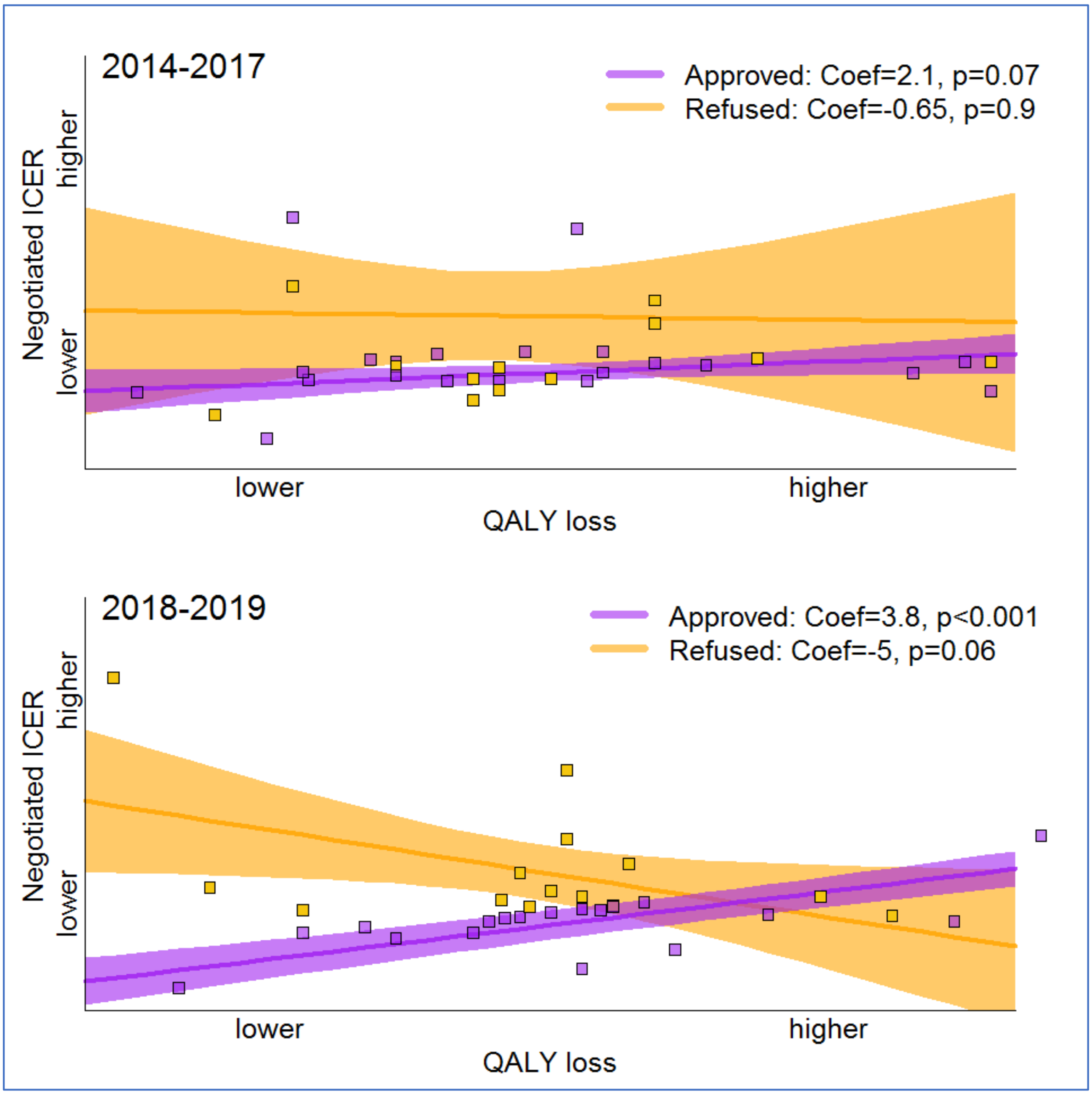

Figure legend: Plots for corresponding regression lines for the relationship between severity (QALY loss) and cost-effectiveness (negotiated ICER). Some observations have been omitted to keep information about individual decisions confidential. Coefficients (95% confidence intervals) were calculated based on all observations.

© 2022 Tranvåg EJ et al. *JAMA Network Open.*

eReferences

1. Magnussen, J., Aserud, M., Granaas, T., Magelssen, M., Syse, A., Celius, E.G., et al. På ramme alvor - Alvorlighet og prioritering. 2015.
2. Claxton, K., Martin, S., Soares, M., Rice, N., Spackman, E., Hinde, S., et al. Methods for the estimation of the NICE cost effectiveness threshold. 2013;

© 2022 Tranvåg EJ et al. *JAMA Network Open.*
